# Paralysis of the Stomach

**Published:** 1876-07

**Authors:** P. H. Hayes


### PARALYSIS OF THE STOMACH.

P. H. HAYES, M. D.

A lady past middle life comes to me regularly for treatment, and as a part of her history tells me she has attacks of vomiting of quantities of bile and thick mucus, every week or two, attended by great prostration, headache, chilliness and general exhaustion. After being plied with stimulants and restoratives, she gradually rallies and is in a day or two herself again.

Another lady tells me a very similar story, except that her attacks come on oftener and are much less severe. She vomits like the other great quantities of bile, at first green and afterward yellow, with a great quantity of thick ropy mucus.

It is not many years ago that I learned to set down all cases of this kind as cases of partial paralysis of the pneumogastric nerves, attended by partial paralysis of the stomach, heart, lungs and voice, all which organs are involved by being supplied by these nerves.

In the two cases above, I set myself to work to find out what it is that brings my patients to this sort of crisis about once in so often, and which appears to them so mysterious. In the first case, I find on close inquiry, that it is morphine, and this patient will readily admit where the bane comes from. My second case, on very careful investigation, turns out to be a great lover of tea and coffee. She drinks three cups of tea at each meal, and very strong, and sometimes carries stimulation with tea to the verge of intoxication by adding to them a "*delicious cup of coffee.*" This patient is thoroughly deluded. She says she takes only a very little black tea, and that this "*does her good,*" that she could not get along without. She insists that her case is *very* "peculiar," and consists in a remarkable overflow of bile "about once in so often," and insists that I will have to give it up yet; that this is her natural peculiarity, and when her attacks come on she says, "I told you so, doctor, it's my *liver.*" And so she very angrily repels any suggestion that tends to clear up the character of her liver and put the blame where it truly belongs. Indeed, her liver has a very bad reputation with herself and in her own house, and I half suspect she is willing to have it go so if she could only in this way divert the attention of the doctor from her precious cups.

Only yesterday morning Mrs. Brown sat down to the table a little late, and called to Bridget for her *tea.* In it came and the instant Mrs. Brown tasted it she calls Bridget to take away the "disgusting slops," it was so weak she could not taste a bit of tea in it. And she further added aside to a boarder, "if anything did make her mad it was to have such stuff as that offered her after it had been filled up over and over again and no strength to it." A graduate from the Inebriate Assylum sitting at the next table called out to Mrs. Brown, "now you know how to pity us poor devils; we don't want our whisky a bit worse than you want your strong tea. You are the same slave with a different master."

A young married gentleman comes to my office with very weak digestion and a very pale face, and on close inquiry I trace all the characters of partial paralysis of the stomach to a prolonged and hopeless domestic sorrow. Here I was able to exclude all other causes, and this is no doubt the true one. In this case there is palpitation, a feeble voice, a disposition to talk hurriedly, and inability to bear anything upon the stomach except the simplest food and in the smallest quantity, and this is attended often by colic and flatulency.

John Hunter died of Angina Pectoris, another form of paralysis of the pneumogastric nerves, and the final fatal attack was superinduced by anger, as had many less violent attacks which had in the course of some years preceded this.

One of the very commonest forms of paralysis of the stomach is the sick headache to which so many persons are subject. I enquired of Joseph, a young lad, how his mother was to-day, and Joseph replied very frankly, she went to a party night before last and she had the sick headache the next day. Now this form of paralysis of the stomach comes on from over-loading with rich food and dainties. Some people I know, bring on this form of paralysis at very regular intervals of about once a month, and they do it by excess at the table. Their continued habit is to eat of rich food beyond the wants of nature, and the continually overtasked stomach sets up this periodical form of rebellion. The eye is made to receive and enjoy the ordinary light of day, but if we gaze at the sun we produce a temporary partial paralysis of the retina, and when we turn away all around is dark. So the sun-light from the snow blinds the eyes, and for a few minutes renders all the house dark as we enter.

But many professional gentlemen and teachers and professors of both sexes are liable to paralysis of the stomach of a partial character from brain-work, mental anxiety and confinement, though their diet may be of the most unexceptionable quality and quantity.

All forms of partial paralysis of the stomach are attended by a similar partial paralysis of the heart, and this gives rise to palpitation and abdominal pulsation or throbbing at the pit of the stomach. A patient of mine last year consulted me for disease of the heart as he supposed. On putting my ear to the chest, I heard the peculiar beating characteristic of some narcotic, and I immediately found that he was an inveterate coffee drinker. This habit had brought on a continued partial paralysis of the stomach, and through the pneumogastric a partial paralysis of the heart. I urged him to leave off his coffee. He said he would rather I would take away half his food. In less than a year this man died, really from paralysis of the stomach. Literary men are peculiarly tempted to goad a partialy paralyzed stomach with tea and coffee. With business men coffee and the cigar. De Balzac, Frederick Soulier, Charles de Bernard, Henry Boyle, Eugene Sue, Count Alfred D'Orsay, all died in the prime of life, in the meridian of their fame, from paralysis of the stomach superinduced by the cigar and coffee.

I have elsewhere in the BISTOURY said that the digestive organs are a man's foundation; that the stomach is the fountain of the blood, and that "the blood is the *life* of the flesh," and it follows that to paralyze the stomach is by so much to paralyze all the powers and actions of life. It is in the stomach that life truly ebbs and flows. This is the "golden bowl" that is finally broken *before* "the wheel at the cistern stops."

